# Association of late-life body mass index with the risk of Alzheimer disease: a 10-year nationwide population-based cohort study

**DOI:** 10.1038/s41598-022-19696-2

**Published:** 2022-09-12

**Authors:** Su Hwan Cho, Minseol Jang, Hyorim Ju, Min Ju Kang, Jae Moon Yun, Jae Won Yun

**Affiliations:** 1 Department of Family Medicine, Seoul National University Hospital, 101, Daehak-ro, Jongno-gu, Seoul, 03080 Republic of Korea; 2 Department of Family Medicine, Dankook University Hospital, 201, Manghyang-ro, Dongnam-gu, Cheonan, Chung Nam 31116 Republic of Korea; 3 Department of Neurology, Veterans Health Service Medical Center, Seoul, Republic of Korea; 4 Veterans Health Service Medical Center, Veterans Medical Research Institute, Jinhwangdo-ro 61-gil 53, Gangdong-gu, Seoul, 05368 Republic of Korea

**Keywords:** Neurology, Risk factors

## Abstract

Existing data for the association between late-life body mass index (BMI) and the risk of Alzheimer’s disease (AD) in the underweight population are limited with conflicting results. A large population-based cohort study of 148,534 individuals aged ≥ 65 years who participated in the national health screening program from 2002 to 2005 was performed using the Korean National Health Insurance Service-Senior cohort database 2006–2015. The risk of AD according to BMI category (kg/m^2^) in Asians was evaluated using a multivariable Cox regression model, after adjustments for age, sex, lifestyle, low-income status, and comorbidities. To evaluate the association between BMI and AD risk, the underweight population was further subdivided according to the degree of thinness. During the 10-year follow-up period, 22,279 individuals developed AD. Relative to the normal-weight population, the estimated adjusted hazard ratio (HR) for incident AD in the underweight, overweight, and obese populations was 1.17 (95% confidence interval [CI], 1.09–1.24), 0.90 (0.87–0.93), and 0.83 (0.80–0.85), respectively. In the underweight population, AD risk increased as the degree of thinness increased (*p* for the trend, < .001). Late-life BMI showed a significant inverse relationship with AD risk, especially in the underweight population. Public health strategies to screen for AD more actively in the underweight population and improve their weight status may help reduce the burden of AD.

## Introduction

Dementia is a clinical syndrome characterized by progressive cognitive decline, behavioral disturbances, and significant interference in the ability to maintain activities of daily living^1^. Approximately 9.9 million individuals are estimated to develop dementia each year, and the number of people with dementia worldwide is predicted to increase to 75 million by 2030. Alzheimer’s disease (AD) is the most common form of dementia and may contribute to 60%-70% of all dementia cases^2^. The principal risk factor for AD is age, and the prevalence of AD has been reported to increase dramatically with age in the US: 5.3% in people aged 65–74 years and 34.6% in those aged 85 years or older^3^. However, AD is not a normal part of aging, and older age alone is not sufficient to cause AD^4^.

Addressing multiple modifiable risk factors for dementia, such as physical activity, smoking, social activity, blood pressure, or diet, can prevent or delay up to 40% of all dementia cases^5^. Among these modifiable risk factors, body mass index (BMI) has a complex effect on the incidence of dementia according to age. A high BMI in mid-life has been relatively well-demonstrated to increase the risk of AD, and many studies have shown consistent results in this regard^6,7^. However, in contrast to the findings for mid-life BMI, the late-life BMI as a continuous variable showed the inverse association with the AD risk in a recent meta-analysis study^8^. The exact underlying mechanisms are still unclear but one plausible explanation for this might be due to the fat-derived hormone leptin^6^. A previous study showed that higher plasma leptin levels were associated with a lower risk of AD^9^, suggesting leptin may play a role in reducing the production of amyloid beta (Aβ)^10^, diminishing extracellular Aβ^11^, and inhibiting the production of hyperphosphorylated tau which are the key pathological features of AD^11,12^.

However, previous longitudinal studies comparing the risk of AD between low and non-low BMI in late-life showed inconclusive results, which were primarily attributed to the differences in the criteria for low BMI between studies and the small number of participants included in the studies^6,8,13^. Although obese individuals (BMI ≥ 30 kg/m^2^) have been reported to show a significantly lower AD risk than those with lower BMI, these studies did not include an adequate number of participants to provide sufficient evidence to prove the generalization that obesity reduces the risk of AD. In addition, since these studies did not include Asians^14–16^, the applicability of these results to Asian participants with different BMI criteria is not clear.

Therefore, we conducted this nationwide cohort study with sufficient late-life participants to confirm the relationship between late-life BMI and AD risk. We also used a population-based representative sample from the Republic of Korea to determine whether the late-life weight state according to the Asia–Pacific BMI classification is associated with the incidence of AD.

## Methods

### Data source and study population

The Korean National Health Insurance Service (NHIS), which is a single compulsory social medical insurer operated by the government, has established a public database called the National Health Information Database (NHID). The NHID contains all records of healthcare utilization (including information on diagnosis and prescription records), the eligibility database (including sociodemographic variables), and the national health screening database^17^. The national health screening program consists of a questionnaire about previous history, family history, lifestyle, anthropometric measurements, and laboratory tests, and is provided biennially to all adults older than 40 years^18^. The NHIS-Senior database is composed of a 10% randomly sampled group of the entire elderly population aged ≥ 60 years in the NHID in 2002^19^. All individuals in the NHIS-Senior cohort were followed up retrospectively from 2002 to 2015, except for those who were not eligible for national health insurance.

**Figure 1:**
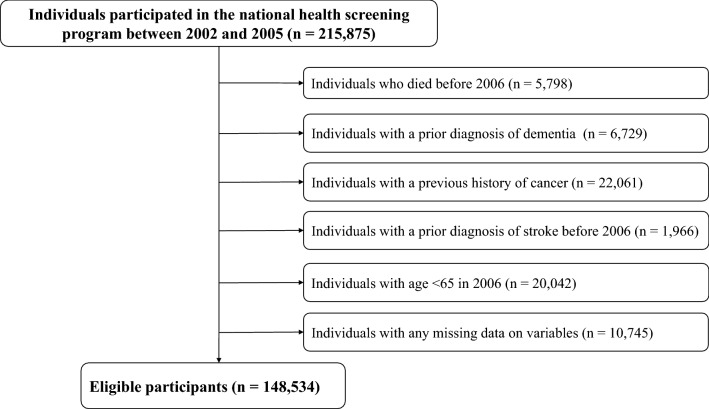
Flow chart of the study population.

We collected data from the NHIS-Senior database for all individuals who participated in the national health screening program from 2002 to 2005. Among the 215,875 participants, we excluded 5798 individuals who died before the index date and 6729 individuals who had received a diagnosis of any type of dementia before the index date. In addition, we excluded 22,061 individuals who had been diagnosed with any type of cancer and 1966 individuals who had a history of stroke before the index date. We then excluded 20,042 participants aged < 65 years in 2006 and 10,745 individuals with missing variables. The final study population consisted of 148,534 individuals (Fig. 1). All participants were followed from the index date, January 1, 2006, to the date of AD diagnosis or December 31, 2015, whichever was earlier.

The Institutional Review Board of the Veterans Health Service Medical Center (Seoul, Republic of Korea) approved this study (IRB no. BOHUN 2021-01-059-001), and waived the requirement for obtaining written informed consent because the NHID provides anonymized and de-identified data. All research was performed in accordance with the 1964 Declaration of Helsinki and its later amendments.

### Outcome and covariable definitions

AD was diagnosed on the basis of International Classification of Diseases, 10th revision codes (ICD-10) codes F00 or G30. We defined AD in cases where the diagnosis and prescription of anticholinesterases (donepezil, rivastigmine, and galantamine) or *N*-methyl-d-aspartate (NMDA) receptor antagonists (memantine) were claimed together on the same day^20–22^. To properly claim the prescription of anti-dementia drugs, physicians should document the evidence of cognitive decline according to the following criteria: 1) Mini Mental State Examination (MMSE) score ≤ 26 and 2) either Clinical Dementia Rating (CDR) ≥ 1 or Global Deterioration Scale (GDS) score ≥ 3^23^.

Participants were categorized by BMI (kg/m^2^) as underweight (< 18.5), normal weight (18.5–22.9), overweight (23.0–24.9), or obese (≥ 25.0) using the WHO Western Pacific Region guideline strata^24^. The underweight population was further categorized as showing mild (17.0–18.4), moderate (16.0–16.9), or severe (< 16.0) thinness^25^. Participants responded to questionnaires regarding their past medical history and health behaviors, such as current smoking, current alcohol drinking, and regular exercise (at least 1 time per week) in the national health screening program. Because health insurance premiums are determined by income level or holding property in the NHIS, we defined the low-income population as individuals whose health insurance premiums were less than the lowest decile for the insured or who were medical aid beneficiaries.

Comorbidities such as hypertension, diabetes, and dyslipidemia were defined by prescription of medication for the disease using the respective ICD-10 codes (I10–13 and I15 for hypertension, E11–14 for diabetes, and E78 for dyslipidemia) at least 2 times per year before the index year or if the respective diagnostic criteria were met (systolic blood pressure ≥ 140 mmHg or diastolic blood pressure ≥ 90 mmHg for hypertension; fasting blood glucose ≥ 126 mg/dL for diabetes; and total cholesterol ≥ 240 mg/dL for dyslipidemia) in the results of the national health screening program. Cardiovascular disease (CVD) was identified based on the answers to the self-reported questionnaire for a physician’s diagnosis of heart disease in the national health screening program.

For each participant, the primary outcome was the occurrence of AD between January 1, 2006, and December 31, 2015, and the number of person-years of follow-up was recorded.

### Statistical analysis

The baseline characteristics of the participants were compared according to BMI categories using ANOVA for continuous variables and chi-square test for categorical variables. Data are presented as the mean (standard deviation) or number (%). The AD incidence rates were calculated by dividing the number of events by 1,000 person-years (PY). Cox proportional hazards regression analyses were performed to obtain hazard ratios (HRs) and 95% confidence intervals (CIs) of AD based on baseline BMI categories. The risk of AD was analyzed after adjusting for possible confounding factors. Model 1 was adjusted for age and sex, and Model 2 was additionally adjusted for lifestyle factors (smoking status, alcohol consumption, and regular exercise) and low-income status. Model 3 was further adjusted for a history of hypertension, diabetes, dyslipidemia, and CVD. Stratified analysis was performed by dividing the participants into subgroups according to baseline age group (65–74 or ≥ 75 years), sex, low-income status, current smoking, alcohol consumption, regular exercise, underlying hypertension, diabetes, dyslipidemia, and history of CVD to test interactions between subgroups. In addition, a sensitivity analysis was performed using multiple imputation to additionally deal with missing values based on the method of fully conditional specification. Statistical analyses were performed using the SAS Enterprise Guide (version 7.1; SAS Institute, Cary, NC, USA) and STATA software (MP, version 17.0; StataCorp, College Station, TX, USA), and statistical significance was defined as two-sided *p* < 0.05.

## Results

### Baseline characteristics of the study population

Our study enrolled 148,534 individuals aged 65 years or older, and 22,279 AD events were observed during the 10-year follow-up period. As shown in Table 1, the average age of the underweight population was the highest (mean, 73.8 years), and the proportion of men was higher (51.0%) in the whole population. Average blood pressure and fasting blood sugar and total cholesterol levels increased as BMI increased (P < 0.001). The underweight population included a higher rate of current smokers (27.9%) than other populations. The obese population had the lowest percentage of current alcohol drinkers (23.7%) among all the groups. The proportion of participants who regularly exercised was lower in the underweight population (21.6%) than in the other populations. The underweight population had fewer comorbidities, such as hypertension (46.4%), diabetes mellitus (17.0%), dyslipidemia (13.8%), and cardiovascular disease (2.6%), than the other populations.

**Table 1:** Baseline characteristics of the study population.

	Underweight	Normal weight	Overweight	Obese	
	(BMI < 18.5 kg/m^2^) (n = 6313)	(18.5 ≤ BMI < 23.0 kg/m^2^) (n = 54,493)	(23.0 ≤ BMI < 25.0 kg/m^2^) (n = 37,699)	(BMI ≥ 25.0 kg/m^2^) (n = 50,029)	P value
Age (years)	73.8 (6.5)	71.8 (5.6)	70.7 (5.1)	70.4 (4.8)	< 0.01
**Age by group (n, %)**					< 0.01
75 years <	3702 (58.6)	39,628 (72.7)	30,009 (79.6)	40,953 (81.9)	
75 years $\ge$	2611 (41.4)	14,865 (27.3)	7690 (20.4)	9076 (18.1)	
**Sex (n, %)**					< 0.01
Male	3222 (51.0)	26,796 (49.2)	17,130 (45.4)	18,091 (36.2)	
Female	3091 (49.0)	27,697 (50.8)	20,569 (54.6)	31,938 (63.8)	
BMI (kg/m^2^)	17.4 (1.0)	21.2 (1.2)	24.0 (0.6)	27.2 (2.0)	< 0.01
**Blood pressure (mmHg)**					
Systolic	127.2 (20.1)	131.4 (19.3)	134.1 (18.8)	136.8 (18.6)	< 0.01
Diastolic	77.2 (11.8)	79.3 (11.5)	80.9 (11.3)	82.3 (11.3)	< 0.01
Fasting glucose (mg/dL)	98.0 (32.5)	100.5 (34.4)	102.8 (35.3)	105.4 (35.8)	< 0.01
Total cholesterol (mg/dL)	185.0 (37.3)	196.0 (38.9)	203.0 (38.7)	207.1 (39.1)	< 0.01
Current smoking (n, %)	1759 (27.9)	10,840 (19.9)	5241 (13.9)	4921 (9.8)	< 0.01
Alcohol drinking (n, %)	1763 (27.9)	15,762 (28.9)	10,476 (27.8)	11,832 (23.7)	< 0.01
Regular exercise^a^ (n, %)	1364 (21.6)	16,558 (30.4)	14,029 (37.2)	18,130 (36.2)	< 0.01
Low income^b^ (n, %)	936 (14.8)	7792 (14.3)	4862 (12.9)	6594 (13.2)	< 0.01
**Comorbidities (n, %)**					
Hypertension	2931 (46.4)	30,994 (56.9)	25,151 (66.7)	38,122 (76.2)	< 0.01
Diabetes mellitus	1070 (17.0)	11,973 (22.0)	10,310 (27.4)	16,283 (32.6)	< 0.01
Dyslipidemia	873 (13.8)	13,413 (24.6)	12,502 (33.2)	19,817 (39.6)	< 0.01
CVD^c^	162 (2.6)	1634 (3.0)	1277 (3.4)	2128 (4.3)	< 0.01

Data are presented as the mean (SD) and number (%).

BMI, body mass index; CVD, cardiovascular disease.

^a^Regular exerciser was defined as individuals who exercised at least once per week.

^b^Low income was defined as medical beneficiaries and the lowest decile of health insurance premiums.

^c^CVD was defined as answers to the self-report questionnaire in which heart disease was previously diagnosed by a physician or was being treated.

### Risk of incident AD according to baseline BMI category

**Figure 2:**
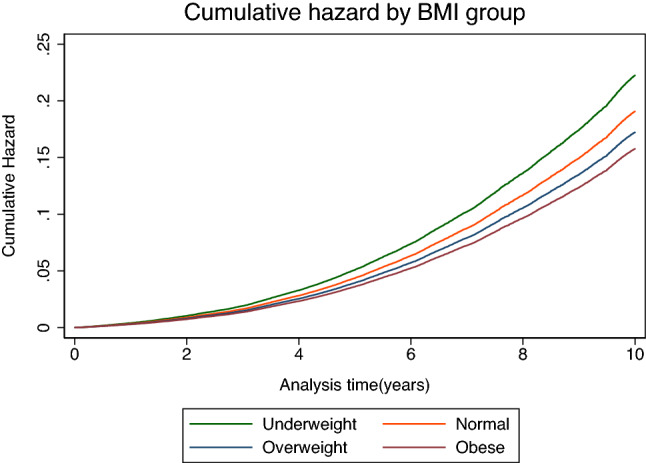
The cumulative adjusted hazard ratio of Alzheimer disease by late-life body mass index categories for 10 years.

Table 2 shows the HRs (95% CIs) of incident AD according to baseline BMI. During the 10-year follow-up period, after adjusting the results for age, sex, smoking status, alcohol consumption, regular exercise, low-income status, hypertension, diabetes, dyslipidemia, and CVD (model 3), the risk of AD significantly decreased as BMI increased (*p* for trend, < 0.001). When the normal-weight population was referenced, the risk of AD increased in the underweight population (adjusted HR in the fully adjusted model = 1.17, 95% CI = 1.09–1.24). The estimated adjusted HR for incident AD in the underweight population relative to the normal-weight population increased as the degree of thinness increased (adjusted HR = 1.13, 95% CI = 1.05–1.22 for mild thinness; adjusted HR = 1.25, 95% CI = 1.09–1.44 for moderate thinness; adjusted HR = 1.24, 95% CI = 1.02–1.51 for severe thinness; *p* for trend, < 0.001). In contrast, the estimated HR for incident AD was inversely associated with increased BMI (adjusted HR = 0.90, 95% CI = 0.87–0.93 for the overweight population and adjusted HR = 0.83, 95% CI = 0.80–0.85 for the obese population). The cumulative hazard for 10 years according to the BMI categories is shown in Fig. 2. Sensitivity analysis performed by multiple imputation on missing values showed almost the same results (adjusted HR in the fully adjusted model = 1.18, 95% CI = 1.11–1.25 for underweight; adjusted HR = 0.91, 95% CI = 0.88–0.94 for overweight; adjusted HR = 0.83, 95% CI = 0.81–0.86 for obesity).

**Table 2:** The incidence and risk of Alzheimer’s dementia by BMI category.

BMI category (kg/m^2^)	Number at risk	AD events	Person-years	Incidence rate (per 1000 person-year)	Estimated hazard ratio (95% confidence interval)			
					Unadjusted	Model 1^a^	Model 2^b^	Model 3^c^
< 18.5	6313	1079	46,677.63	23.12	1.29 (1.21, 1.38)	1.14 (1.07, 1.22)	1.12 (1.05, 1.20)	1.17 (1.09, 1.24)
18.5–22.9	54,493	8601	455,382.67	18.89	Reference	Reference	Reference	Reference
23.0–24.9	37,699	5497	329,287.24	16.69	0.87 (0.84, 0.90)	0.92 (0.89, 0.95)	0.93 (0.90, 0.97)	0.90 (0.87, 0.93)
$\ge$ 25.0	50,029	7102	443,525.00	16.01	0.83 (0.80, 0.85)	0.87 (0.84, 0.89)	0.88 (0.85, 0.91)	0.83 (0.80, 0.85)
p for trend					< 0.001	< 0.001	< 0.001	< 0.001

BMI, body mass index; AD, Alzheimer’s dementia.

^a^Adjusted for age and sex.

^b^Adjusted for variables in model 1 and smoking, alcohol consumption, regular exercise, and low-income status.

^c^Adjusted for variables in model 2 and hypertension, diabetes mellitus, dyslipidemia, and cardiovascular disease.

### Risk of incident AD according to baseline BMI category in subgroups categorized by age, sex, and underlying diabetes

**Figure 3:**
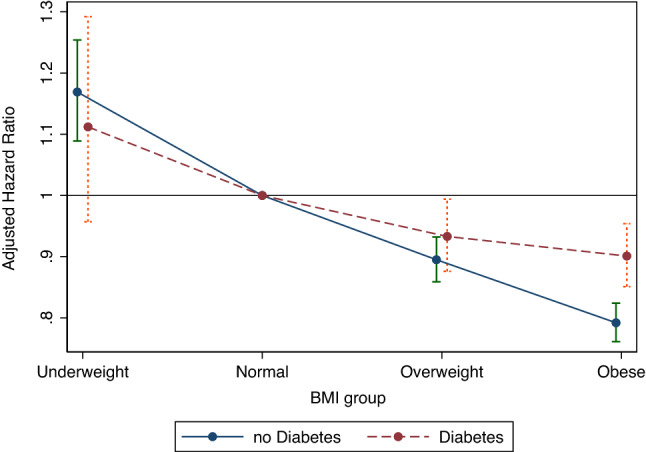
Subgroup analysis of the adjusted hazard ratio for Alzheimer disease by body mass index.

The interaction between BMI category and diabetes status on the risk of AD was significant, and the risk of AD according to BMI in the diabetes-free subgroup showed a greater difference (*p* < 0.001, Fig. 3). No significant interactions between BMI category and age, sex, low-income status, current smoking, alcohol consumption, regular exercise, underlying hypertension, dyslipidemia, and history of CVD were observed in the occurrence of AD (only some data are presented in Table 3).

**Table 3:** Subgroup analysis of adjusted hazard ratio for AD by BMI according to age, sex, and diabetes.

Subgroup	BMI category (kg/m^2^)	AD events	Incidence rate (per 1000 person-year)	Adjusted hazard ratio (95% CI)
**Age, years**				
< 75	$<$ 18.5	501	16.36	1.26 (1.15, 1.38)
	18.5–22.9	4842	13.80	Reference
	23.0–24.9	3473	12.75	0.88 (0.84, 0.92)
	$\ge$ 25.0	4812	12.87	0.82 (0.79, 0.85)
≥ 75	$<$ 18.5	578	35.99	1.08 (0.98, 1.17)
	18.5–22.9	3759	35.95	Reference
	23.0–24.9	2024	35.56	0.95 (0.90, 1.01)
	$\ge$ 25.0	2290	32.90	0.84 (0.79, 0.88)
P for interaction				0.564
**Sex**				
Men	$<$ 18.5	390	17.04	1.11 (1.00, 1.23)
	18.5–22.9	3226	14.74	Reference
	23.0–24.9	1928	13.07	0.92 (0.87, 0.97)
	$\ge$ 25.0	1881	11.89	0.84 (0.79, 0.89)
Women	$<$ 18.5	689	28.96	1.19 (1.10, 1.29)
	18.5–22.9	5375	22.73	Reference
	23.0–24.9	3569	19.64	0.90 (0.86, 0.93)
	$\ge$ 25.0	5221	18.30	0.82 (0.79, 0.86)
P for interaction				0.408
**Diabetes**				
Yes	$<$ 18.5	187	26.01	1.11 (0.96, 1.29)
	18.5–22.9	2147	22.56	Reference
	23.0–24.9	1779	20.52	0.93 (0.88, 0.99)
	$\ge$ 25.0	2845	20.41	0.90 (0.85, 0.95)
No	$<$ 18.5	892	22.59	1.17 (1.09, 1.25)
	18.5–22.9	6454	17.92	Reference
	23.0–24.9	3718	15.33	0.89 (0.86, 0.93)
	$\ge$ 25.0	4257	14.00	0.79 (0.76, 0.82)
P for interaction				< 0.001

BMI, body mass index; AD, Alzheimer’s dementia.

^a^Adjusted for age and sex.

^b^Adjusted for variables in model 1 and smoking, alcohol consumption, regular exercise, and low-income status.

^c^Adjusted for variables in model 2 and hypertension, diabetes mellitus, dyslipidemia, and cardiovascular disease.

## Discussion

In this large nationwide population-based cohort study, BMI was significantly associated with an increased risk of incident AD in an inverse linear relationship, and this trend was maintained even when the underweight population was subdivided. In comparison with normal-weight participants, those who were underweight, overweight, and obese showed 1.17-, 0.90-, and 0.83-fold risk of AD in the fully adjusted model, respectively.

Our study showed clear evidence for the association between lower late-life BMI and the increased risk of AD and has the advantage of extending these findings to the underweight population. Previous cohort studies did not confirm definitive results as to whether the risk of AD increased in the underweight group compared to the normal-weight group because the number of participants was limited^14,26,27^. In addition, among the previous studies that investigated risk factors for AD in Asians, few have been conducted on the basis of late-life BMI^28^. Since our study was a nationwide representative cohort study and included enough underweight individuals, our findings provide more robust evidence of the association between underweight status and AD incidence in the general population. In addition, an inverse linear relationship appeared when the risk of AD was measured by dividing the late-life BMI group using the Asian-Pacific classification^24^ which is different from the Western classification.

Previous studies have attempted to reveal the association between late-life BMI and Aβ and tau, which are known to play key roles in the pathogenesis of AD. A recent study showed that a higher late-life BMI was associated with higher levels of cerebrospinal fluid (CSF) Aβ42 and lower levels of t-tau and t-tau/Aβ42^29^, which have superior accuracy for the detection of AD and could indirectly indicate the extent of AD pathology in the brain^30,31^. In addition, a lower load of brain cortical Aβ and larger brain volumes of AD-vulnerable regions were associated with higher late-life BMI, and the longitudinal data suggested that individuals with higher late-life BMI had less cognitive decline^29^. Likewise, several studies that measured the association between the accumulation of brain cortical Aβ and BMI in the elderly with normal cognitive function repeatedly proved the association between high BMI and low Aβ burden^32,33^. Considering the association between late-life BMI and key molecules involved in the pathogenesis of AD, it can be inferred that a certain mechanism related to late-life BMI affects the risk of AD.

The fat-derived hormone leptin has been identified as a key biomarker to explain the association between late-life BMI and the risk of developing AD. Leptin is a hormone derived from adipocytes that is secreted in proportion to the amount of adipocytes in the body, with high circulating plasma levels in obesity and low circulating plasma levels in states of starvation^34^. Leptin reduces the production of Aβ from amyloid precursor proteins by inhibiting the γ-secretase complex^10^ and diminishes extracellular Aβ by driving Aβ uptake into cells^11^. Leptin inhibits the production of hyperphosphorylated tau that forms neurofibrillary tangles, which are the key pathological features of AD^11,12^. The correlation between plasma leptin levels and body fat is known to persist with age and appear in older adults^35^. In addition, previous observational studies also support the notion that leptin may be protective against dementia, since low plasma leptin levels in late-life are associated with worsening cognitive decline and increased dementia risk^9,36^. Collectively, these findings suggest that a low leptin level state due to low late-life BMI can reduce its neuroprotective effects, which would lead to a downward cascade of worsening AD pathology and further weight loss^37^. Our study showed that lower BMI was associated with and increased risk of AD, which may have contributed to decreased leptin levels and the corresponding deterioration of neuroprotective effects.

In our subgroup analyses, an interaction was observed in which the underlying diabetes status affected the risk of AD according to late-life BMI, with a greater difference in the risk of AD according to the BMI category in the non-diabetic subgroup. This suggests that the neuroprotective effect of leptin, which increases with increasing body weight, decreases in individuals with diabetes. One plausible explanation for this is that diabetes attenuates the neuroprotective effects of leptin by increasing leptin resistance^38^. Although the exact mechanism has not been elucidated, diabetes increases resistance to leptin, which is presumed to interfere with the action of leptin^39^. In fact, a previous study showed that the late-life obese group with metabolic syndrome, which is known to increase leptin resistance similar to diabetes, had an increased AD risk than a normal-weight group without metabolic syndrome^40^. Thus, the protective action to reduce the risk of AD by hyperleptinemia caused by high late-life BMI may be modulated by other factors. Further studies are required to elucidate this hypothesis.

Thus, our study is noteworthy in that it clearly presented the relationship between late-life BMI and the risk of AD in a nationwide population-based cohort. As the BMI category changed from obese to underweight, the risk of AD increased in a weight-dependent manner, and this trend was maintained even when underweight group was subdivided. These results suggest that improving public nutritional status to prevent weight loss in older adults may be a key strategy for reducing the incidence of AD. A previous study showed that weight loss in older adults was associated with the development of AD regardless of the basal BMI status, which supports this view^41^. Therefore, policy support to manage the weight of older adults through active nutritional interventions may be helpful in preventing the occurrence of AD. Further studies are needed to determine whether increasing the weight of the elderly reduces the incidence of AD.

### Limitation

Our study had several limitations. First, the diagnosis of AD was made using ICD-10 codes and not individual clinical diagnoses. To overcome this, the time of AD onset was defined as the simultaneous occurrence of the ICD-10 codes for AD and anti-dementia drug prescriptions that must have the patient’s result of the MMSE and the CDR or GDS to claim reimbursement^23^. Second, the levels of biomarkers such as leptin or genetic factors such as APOE could not be measured because they were not included in the national health screening program. Third, although our study targeted a very large population group, the participation rate for the national health screening program in the early 2000s was less than half; therefore, we could not include more participants. Selection bias may have occurred because only those participating in the national health checkup program were included. Fourth, a recent study reported that sarcopenia is associated with AD occurrence. In this study, of the two components of sarcopenia, muscle function and lean muscle mass, the former was associated with incident AD, while the latter was not^42^. Since our study was conducted based on BMI, it did not accurately reflect the body fat mass or lean muscle mass of the elderly participants. Further investigation regarding the incidence of AD seems necessary, considering both BMI and components of sarcopenia in the late-life population. Fifth, the difference in AD risk according to late-life BMI may have been underestimated. In another study that followed 10-year mortality among Koreans aged 65 years or older, the lower the baseline BMI, the higher the mortality rate^43^ which implied a higher chance of dying before AD develops. Finally, our study could not prove a causal relationship between late-life BMI and the incidence of AD because many possible confounding factors affecting the incidence of AD that cannot be identified in this database were not considered. Nevertheless, this study has value as the largest population-based study analyzing late-life BMI and AD over a 10-year follow-up. It is also meaningful because it more accurately assesses the risk of AD in Asians, including sufficient underweight populations.

## Conclusion

In conclusion, the severity of underweight as defined by late-life BMI was associated with a higher risk of AD incidence in a weight-dependent manner, even in an underweight population. Strategies to gain weight in later life can help pave the way for reducing the burden of AD.

## Data Availability

The data that support the findings of this study are available from National Health Information Database but restrictions apply to the availability of these data, which were used under license for the current study, and so are not publicly available. Data are however available from the authors upon reasonable request and with permission of National Health Insurance Sharing Service. (https://nhiss.nhis.or.kr).

## Abbreviations

AD: Alzheimer’s disease
Aβ: Amyloid beta
BMI: Body mass index
CVD: Cardiovascular disease
CSF: Cerebrospinal fluid
CDR: Clinical dementia rating
CIs: Confidence intervals
GDS: Global Deterioration Scale
HRs: Hazard ratios
ICD-10: International Classification of Diseases, 10th revision codes
MMSE: Mini Mental State Examination
NHIS: National Health Insurance Service
NHID: National Health Information Database
NMDA: *N*-Methyl-d-aspartate
PY: Person-years
